# Large-scale avian vocalization detection delivers reliable global biodiversity insights

**DOI:** 10.1073/pnas.2315933121

**Published:** 2024-08-06

**Authors:** Sarab S. Sethi, Avery Bick, Ming-Yuan Chen, Renato Crouzeilles, Ben V. Hillier, Jenna Lawson, Chia-Yun Lee, Shih-Hao Liu, Celso Henrique de Freitas Parruco, Carolyn M. Rosten, Marius Somveille, Mao-Ning Tuanmu, Cristina Banks-Leite

**Affiliations:** ^a^Department of Life Sciences, Imperial College London, London W12 7TA, United Kingdom; ^b^Norwegian Institute for Nature Research, Trondheim 7034, Norway; ^c^Department of Forestry and Resources Conservation, National Taiwan University, Taipei 106, Taiwan; ^d^Mombak, São Paulo 04547-000, Brazil; ^e^Division of Biosciences, University College London, London WC1E 6AE, United Kingdom; ^f^Biodiversity Research Center, Academia Sinica, Taipei 11529, Taiwan; ^g^Independent Researcher, Brazil

**Keywords:** biodiversity, machine learning, acoustics, bioacoustics, birds

## Abstract

Tracking biodiversity and its dynamics at scale is essential if we are to solve global environmental challenges. Detecting animal vocalizations in passively recorded audio data offers an automatable, inexpensive, and taxonomically broad way to monitor biodiversity. However, the labor and expertise required to label new data and fine-tune algorithms for each deployment is a major barrier. In this study, we applied a pretrained bird vocalization detection model, BirdNET, to 152,376 h of audio comprising datasets from Norway, Taiwan, Costa Rica, and Brazil. We manually listened to a subset of detections for each species in each dataset, calibrated classification thresholds, and found precisions of over 90% for 109 of 136 species. While some species were reliably detected across multiple datasets, the performance of others was dataset specific. By filtering out unreliable detections, we could extract species and community-level insight into diel (Brazil) and seasonal (Taiwan) temporal scales, as well as landscape (Costa Rica) and national (Norway) spatial scales. Our findings demonstrate that, with relatively fast but essential local calibration, a single vocalization detection model can deliver multifaceted community and species-level insight across highly diverse datasets; unlocking the scale at which acoustic monitoring can deliver immediate applied impact.

Biodiversity plays a crucial role in food security, disease dynamics, human well-being, and more (1). However, the vast number of species and the complexity of their interactions make tracking biodiversity and understanding how it is impacted by anthropogenic activities a challenge (2). Reliable, scalable, and taxonomically diverse biodiversity monitoring is therefore essential if we are to thrive sustainably as a society.

Traditional biodiversity surveys are time consuming and require niche expertise. However, declines in cost and increased accessibility of robotics platforms and electronic sensors have transformed our ability to survey ecosystems at larger scales (3). Using autonomous sensing technologies, scientists have tracked cetaceans in the Pacific from drones (4), mammals in the Serengeti with camera traps (5), and bats across London from ultrasonic microphones (6). However, the machine learning models used in each of these cases were trained on manually labeled subsets of data from the study system of interest. “Plug-and-play” approaches which convert raw field sensor data into reliable species community insight across diverse ecosystems, without retraining models, have not been demonstrated to work reliably to date.

Due to the diversity of species and their behaviors, we are unlikely to ever develop a single technology to monitor all biodiversity in all ecosystems. Nevertheless, detection and classification of animal vocalizations in long-term acoustic recordings is a promising approach in its ability to scale well temporally, spatially, and taxonomically. Bird vocalizations in particular have been recorded by hobbyists and scientists for decades culminating in rich libraries of annotated data which span the globe (7). Classification models have been trained on these libraries (8, 9) and some studies have evaluated the performance of these models on single datasets (10). However, no studies have looked at the performance of vocalization classification models when applied to multiple large acoustic datasets collected across diverse ecosystems.

In this study, we investigated the following: i) how reliably can we monitor bird communities across diverse datasets using a single vocalization detection model and ii) what biodiversity insight could such an approach deliver?

## Results

**Fig. 1.**
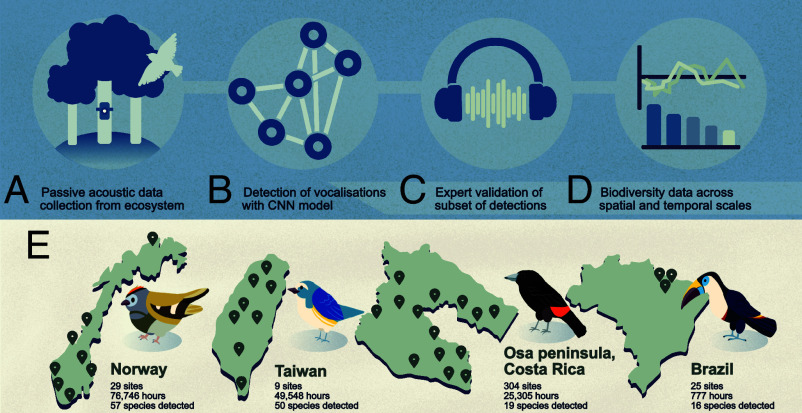
Study overview. (*A*) We recorded 152,376 h of acoustic data from ecosystems. (*B*) BirdNET, a state-of-the-art convolutional neural network model, was used to detect and classify bird vocalizations. (*C*) Experts manually labeled a subset of the detections for each species in each dataset. (*D*) We used filtered detections to derive reliable avian biodiversity insight across spatial and temporal scales. (*E*) Approximate sampling locations across Norway, Taiwan, the Osa Peninsula in Costa Rica, and State of Pará in Brazil. Species depicted are Goldcrest (Norway), Red-flanked Bluetail (Taiwan), Scarlet-rumped Tanager (Costa Rica), and White-throated Toucan (Brazil).

We collected 152,376 h of passively recorded acoustic data from temperate forests across Norway (76,746 h), tropical and subtropical forests across Taiwan (49,548 h), diverse tropical landscapes across the Osa Peninsula in Costa Rica (25,305 h), and tropical forests in the Amazon, State of Pará, Brazil (777 h) (Fig. 1 *A* and *E*). We detected bird vocalizations in the audio data using an open-source convolutional neural network (CNN) model, BirdNET (8), with the geographic species filter enabled. In total, the model outputted 627,995 detections of 379 species with model classification scores of above 0.80 (Fig. 1*B*). To ensure that we had enough verification data, we only considered species with over 50 detections, leaving 625,113 detections from 136 species (Fig. 1*C*).

**Fig. 2.**
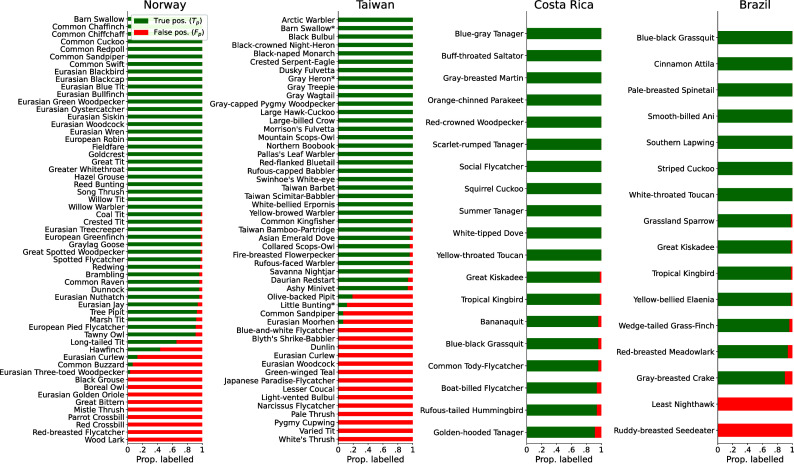
BirdNET was highly precise for many species across diverse datasets. An expert manually labeled 50 BirdNET detections of each species in each dataset to calibrate classification thresholds and measure precision (T_p_/[T_p_+F_p_], where T_p_ and F_p_ are true and false positives, respectively). We found 43/57 species in Norway, 33/51 in Taiwan, 19/19 in Costa Rica, and 14/16 in Brazil were detected with over 90% precision. Calibrated thresholds and model performance varied across species and datasets, suggesting that expert validation must be repeated for new deployments. Asterisks denote species with low numbers of detections (once optimal BirdNET thresholds were applied).

We listened to 50 random detections for each species from each dataset and labeled them as true positives (T_p_) or false positives (F_p_) to measure model performance (c. 20 to 30 min labeling effort per species, Fig. 1*C*). Measuring recall was intractable on such large datasets (10). We calibrated classification thresholds for each species in each dataset (11) and found in Norway 43/57, Taiwan 33/51, Costa Rica 19/19, and Brazil 14/16 species all reached over 90% precision—i.e., the model very rarely outputted false positive detections for these species (Fig. 2). Full avian communities in the sampled ecosystems are far larger than the subsets that BirdNET detected, but the discrepancy was particularly stark in Brazil and Costa Rica—likely due to long-standing geographical and taxonomic biases in the training datasets used by BirdNET (9).

Seven species appeared in two datasets. Model precision for five species was consistent across datasets: Blue-black Grassquits, Great Kiskadees, and Tropical Kingbirds in Brazil and Costa Rica, and Barn Swallows and Eurasian Curlews in Norway and Taiwan. However, while detections of Common Sandpipers and Eurasian Woodcocks were reliable in Norway (100% precision), detections of the same two species in Taiwan were highly unreliable (precisions of 7% and 0%, respectively). Inconsistent performance across datasets might be explained by varying dialects, microphones, experts performing the labeling, geophony, anthropophony, and more, indicating that model performance must be recharacterized for each new deployment of acoustic sensors.

**Fig. 3.**
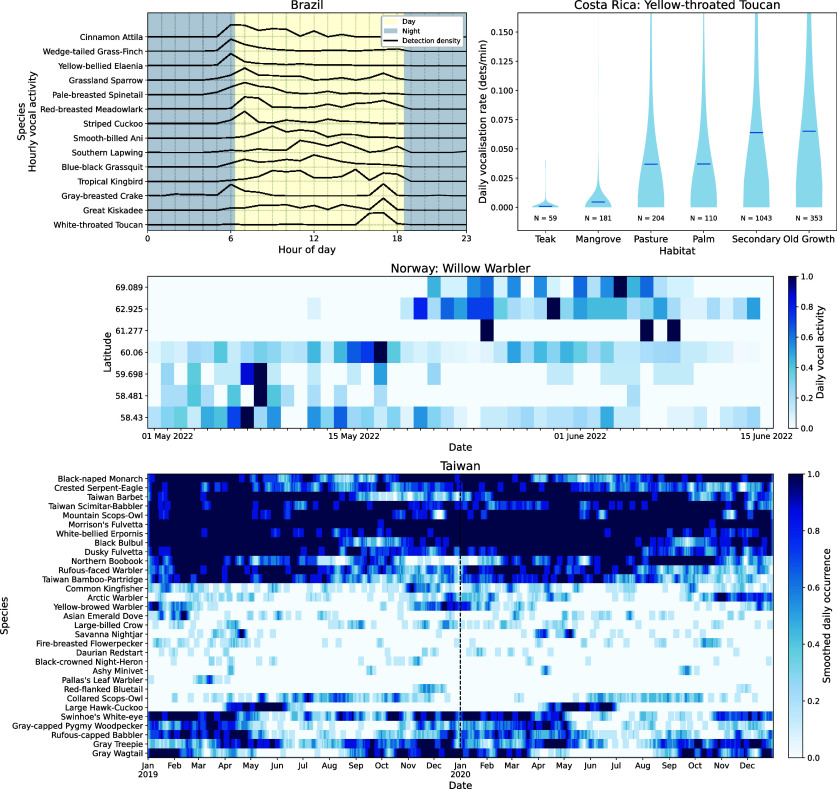
Acoustic data delivered species and community insight at a range of spatial and temporal scales. In Brazil, diel detection patterns varied between species which were more vocal at dawn, throughout the day, or at dusk. On the Osa Peninsula in Costa Rica, we found the rate of detected vocalizations for the Yellow-throated Toucan was highest in old-growth and secondary forests (means shown, *y*-axis limited by 90th percentile of data). In Norway, the Willow Warbler’s spring northward migration through the country was reconstructed. Across 2 y in Taiwan, there were complex temporal dynamics from breeding and wintering migratory birds as well as resident species (species ordered by clustering occurrence data and dotted line demarcates years).

Considering only detections from species with over 90% precision and over 20 detections in each dataset (once optimal BirdNET thresholds were applied), we extracted biodiversity insight across varied spatiotemporal scales and taxonomic resolutions (Fig. 3). In the Brazilian Amazon, diel vocal activity varied between species which were more vocal at dawn (e.g., Pale-breasted Spinetail), during the day (e.g., Blue-black Grassquit), and at dusk (e.g., Great Kiskadee) (Fig. 3*A*). On the Osa Peninsula in Costa Rica, we found daily vocalization rate of the Yellow-throated Toucan varied across habitats with the most frequent vocalizations detected in old-growth and secondary forests, likely indicating habitat-driven variations in population sizes or behaviors (Fig. 3*B*). Across the temperate forests of Norway, we saw the northward movement of the migratory Willow Warbler throughout spring (Fig. 3*C*). In the forests of Taiwan, we found complex temporal occurrence patterns across a 2-y period, from species which visited Taiwan to breed (e.g., Large Hawk-Cuckoo), those which wintered in the country (e.g., Yellow-browed Warbler), to those which were endemic and vocalising year-round (e.g., Taiwan Bamboo-Partridge) (Fig. 3*D*). While we have showcased known phenomena, we also supply the full lists of reliable detections in each dataset for others to explore in the accompanying data.

## Conclusion

We demonstrated that a single vocalization classification model delivered reliable monitoring for many bird species across four large and diverse datasets. However, predictions were not perfect, and only small subsections of full species communities were detectable, especially in Costa Rica and Brazil which remain underrepresented in global libraries of avian vocalizations. Nonetheless, with relatively fast yet essential local calibration, fine resolution and taxonomically broad biodiversity insight could still be unlocked for many species on small and large temporal and spatial scales in all datasets. If training datasets are able to grow in size and accessibility while addressing systematic taxonomic and geographic biases, the performance of machine learning models will continue to improve (9), unlocking further opportunities for fully autonomous acoustic monitoring to be deployed at scale and deliver impact around the world (12).

## Materials and Methods

### Vocalization Detection Model.

BirdNET (8) was used to detect vocalizations. Location data were provided to BirdNET to filter for only species expected at each recorder [based on eBird (13) observations].

### Calibrating Classification Thresholds.

To determine optimal classification thresholds (11), we measured precision using thresholds between 0.80 and 0.99, inclusive. The optimal value was chosen as the lowest threshold that achieved 90% precision. For all results presented, we filtered detections using independent calibrated classification thresholds for each species in each dataset.

## Supplementary Material

Appendix 01 (PDF)

## Data Availability

Code and data used to reproduce figures and results presented in this manuscript are freely available on Zenodo codes 8338721 (14) and 8340251 (15).
