# Supplementary material for: Large-scale avian vocalization detection delivers reliable global biodiversity insights
Source: Proc Natl Acad Sci U S A. 2024 Aug 6;121(33):e2315933121. doi: 10.1073/pnas.2315933121 (PMC11331061; doi:10.1073/pnas.2315933121)
Supplement: Supplementary file 1 — Appendix 01 (PDF) [file pnas.2315933121.sapp.pdf]

Extended Methods for "Large-scale avian vocalisation detection delivers reliable global biodiversity insights"

Sarab S. Sethi*, Avery Bick, Ming-Yuan Chen, Renato Crouzeilles, Ben V. Hillier, Jenna Lawson, Chia-Yun Lee, Shih-Hao Liu, Celso Henrique de Freitas Parruco, Carolyn Rosten, Marius Somveille, Mao-Ning Tuanmu, Cristina Banks-Leite
* sarab.sethi@imperial.ac.uk

Acoustic data

Norway: We surveyed 29 sites in seven regions across the country between March and October 2022. Sites were all in temperate forests, and devices were spaced at least 100m apart. 76,746 hours of single channel audio was recorded continuously at 44.1kHz using Bugg devices[1] (with Knowles SPH0641LU4H-1 MEMS microphones) mounted between 1-2m from ground level. Audio from the Bugg devices was compressed using FFMPEG's implementation of VBR0 MP3 compression (using the libmp3lame library).

Taiwan: We surveyed 9 sites across the country between January 2019 and December 2020. Sites were all in broadleaf forests and spaced at least 17km apart. 49,548 hours of stereo audio was recorded on a schedule of 1 minute every 3 minutes for 24 hours at 44.1kHz using Wildlife Acoustics Song Meter 4 devices mounted between 1-2m from ground level. Audio data was stored in uncompressed WAV format.

Costa Rica: We surveyed 304 sites on the Osa Peninsula between December 2018 and August 2019. Sites were in varying habitats and spaced at least 500m apart. 25,305 hours of single channel audio was recorded continuously between 05:30 and 9:30, 14:30 and 18:30 and 21:00 and 03:00 at 48,000 kHz using Audiomoth[2] devices mounted between 1-2m from ground level. Audio data was stored in uncompressed WAV format.

Brazil: We surveyed 25 sites municipality of Mãe do Rio, in the Pará State in February 2022. Sites were in located in riparian forests and pastures and spaced at least 500m apart. 777 hours of single channel audio was recorded on a schedule of 1 minute every 5 minutes for 24 hours at a sample rate of 48 kHz using 25 Audiomoth[2] devices mounted between 1-2m from ground level. Audio data was stored in uncompressed WAV format.

BirdNET model

We used a convolutional neural network (CNN) model, BirdNET, to detect bird vocalisations[3]. For Norway, we used BirdNET-Lite (https://github.com/kahst/BirdNET-Lite) accessed in November 2022. For Taiwan, Costa Rica, and Brazil, we used the newer BirdNET-Analyzer model v2.3 (https://github.com/kahst/BirdNET-Analyzer) through the birdnetlib Python library. Re-analysing the Norway data with BirdNET-Analyzer would have been ideal, but we lacked the resources to relabel the randomly selected detections, and the conclusions of our study were unlikely to have changed. Location data was provided to BirdNET to filter for only species expected at each recorder (based on eBird observations).

To acquire valid BirdNET inputs we resampled audio to 48kHz and generated non-overlapping three-second spectrograms (window size 8ms, 25% overlap, 64 Mel-scaled frequency bands). In Norway, adjacent detections of the same species were joined together, but in other datasets each three-second detection was kept independently.

Model calibration and performance

For each species in each dataset, we randomly selected 50 detections with BirdNET classification confidences over 0.80. Species with fewer than 50 detections were discounted, leaving 136 species total. Experts familiar with each study region listened to the randomly selected detections and labelled each BirdNET prediction as correct, incorrect, or unsure (e.g., if the call was ambiguous). Conservatively, we denoted unsure labels as false positives.

We then measured precision, *p*, as $p = T_p/(T_p+F_p)$, where T_p and F_p were true and false positives, respectively, using 20 BirdNET classification thresholds spaced evenly between 0.80 and 0.99, inclusive. For each species in each dataset, we determined optimal thresholds by choosing the lowest threshold that achieved 90% precision. For species where 90% precision was not reached using any threshold value, the lowest threshold that maximised precision was chosen.

There was no correlation between calibrated classification thresholds and species precisions in the Taiwan, Norway, and Brazil datasets, but in Costa Rica there was a negative correlation (Pearson's R=-0.60, p=0.007).

We found calibrating classification thresholds did not greatly change the numbers of detections in each dataset. For the 136 species with at least 50 detections in each dataset, without calibration (i.e., using 0.8 as a threshold for all species) there were 625,113 total detections across all datasets, whereas with calibrated thresholds there were 616,046 total detections; only 1.45% of detections were lost.

In downstream analyses (i.e., Figs. 2 and 3), we only considered detections with confidences above the optimal thresholds determined for each species in each dataset.

Species and community analyses

For the following analyses, we used calibrated classification thresholds and only considered species with over 90% precision, and which had at least 20 detections over the calibrated classification thresholds in the dataset.

Brazil: We binned detections by hour of day to measure diel patterns in vocal activity. To normalise the data shown in Fig. 3a, for each species, we divided hourly numbers of detections by the maximum number of detections in a one-hour period. Species were ordered in the visualisation by their peak hour of vocal activity.

Costa Rica: For each day at each site, we counted the number of vocalisations detected for the Yellow-throated Toucan and normalised for sampling effort by dividing by the number of minutes recorded on that day. This allowed us to produce a measure of species vocalisation rate which accounted for imbalances in sampling effort across the sites. Then, to produce Fig. 3b, we grouped vocalisation rates for each day at each site by habitat type using land use maps provided by NASA, created at a scale of 5 × 5 m using Landsat 5 Thematic Mapper (TM) and Landsat 8 Operational Land Imager (OLI).

Norway: We focused on detections of the Willow Warbler between 30/04/2022 and 15/06/2022, and sites were aggregated by region. To show temporal variations in relative vocal activity across regions in Fig. 3c, detections were binned by day, and we divided daily numbers of detections by the maximum number of detections in a day for each region.

Taiwan: We combined detections across all sites and derived daily occurrence (presence/absence) data for each species. For Fig. 3d, we smoothed the daily occurrence data by convolving the binary occurrence time series for

each species with a vector of ones of shape [1x7] (i.e., at the weekly resolution). Species were ordered by clustering the daily occurrence data to group species with similar temporal dynamics across the monitoring period (e.g., breeding migratory, wintering migratory, resident, etc.).

References

1. Sethi, S. S., Ewers, R. M., Jones, N. S., Orme, C. D. L. & Picinali, L. Robust, real-time and autonomous monitoring of ecosystems with an open, low-cost, networked device. *Methods Ecol. Evol.* **9**, 2383–2387 (2018).
2. Hill, A. P. *et al.* AudioMoth: Evaluation of a smart open acoustic device for monitoring biodiversity and the environment. *Methods Ecol. Evol.* **9**, 1199–1211 (2018).
3. Kahl, S., Wood, C. M., Eibl, M. & Klinck, H. BirdNET: A deep learning solution for avian diversity monitoring. *Ecol. Inform.* **61**, 101236 (2021)
